# Nanoparticles With a Specific Size and Surface Charge Promote Disruption of the Secondary Structure and Amyloid-Like Fibrillation of Human Insulin Under Physiological Conditions

**DOI:** 10.3389/fchem.2019.00480

**Published:** 2019-07-30

**Authors:** Alyona Sukhanova, Simon Poly, Svetlana Bozrova, Éléonore Lambert, Maxime Ewald, Alexander Karaulov, Michael Molinari, Igor Nabiev

**Affiliations:** ^1^Laboratoire de Recherche en Nanosciences, LRN-EA4682, UFR de Pharmacie, Université de Reims Champagne-Ardenne, Reims, France; ^2^Laboratory of Nano-Bioengineering, Moscow Engineering Physics Institute, National Research Nuclear University MEPhI, Moscow, Russia; ^3^Department of Membrane Biophysics, Interfaculty Institute of Biochemistry, University of Tübingen, Tübingen, Germany; ^4^Department of Clinical Immunology and Allergology, Sechenov First Moscow State Medical University, Moscow, Russia

**Keywords:** nanomaterials, protein adsorption, quantum dots, proteinopathies, insulin, fibrillation, amyloidosis

## Abstract

Nanoparticles attract much interest as fluorescent labels for diagnostic and therapeutic tools, although their applications are often hindered by size- and shape-dependent cytotoxicity. This cytotoxicity is related not only to the leak of toxic metals from nanoparticles into a biological solution, but also to molecular cytotoxicity effects determined by the formation of a protein corona, appearance of an altered protein conformation leading to exposure of cryptic epitopes and cooperative effects involved in the interaction of proteins and peptides with nanoparticles. In the last case, nanoparticles may serve, depending on their nature, as centers of self-association or fibrillation of proteins and peptides, provoking amyloid-like proteinopathies, or as inhibitors of self-association of proteins, or they can self-assemble on biopolymers as on templates. In this study, human insulin protein was used to analyze nanoparticle-induced proteinopathy in physiological conditions. It is known that human insulin may form amyloid fibers, but only under extreme experimental conditions (very low pH and high temperatures). Here, we have shown that the quantum dots (QDs) may induce amyloid-like fibrillation of human insulin under physiological conditions through a complex process strongly dependent on the size and surface charge of QDs. The insulin molecular structure and fibril morphology have been shown to be modified at different stages of its fibrillation, which has been proved by comparative analysis of the data obtained using circular dichroism, dynamic light scattering, amyloid-specific thioflavin T (ThT) assay, transmission electron microscopy, and high-speed atomic force microscopy. We have found important roles of the QD size and surface charge in the destabilization of the insulin structure and the subsequent fibrillation. Remodeling of the insulin secondary structure accompanied by remarkable increase in the rate of formation of amyloid-like fibrils under physiologically normal conditions was observed when the protein was incubated with QDs of exact specific diameter coated with slightly negative specific polyethylene glycol (PEG) derivatives. Strongly negatively or slightly positively charged PEG-modified QDs of the same specific diameter or QDs of bigger or smaller diameters had no effect on insulin fibrillation. The observed effects pave the way to the control of amyloidosis proteinopathy by varying the nanoparticle size and surface charge.

## Introduction

Proteinopathies are disorders resulting from changes in protein conformation and subsequent aggregation of protein molecules with altered tertiary and quaternary structures, which accumulate in cells and internal environment of the body (Dobson, 2003). These aggregates, particularly their small-sized intermediate forms, have been found to provoke cell membrane oxidation, interfere with ion homeostasis, the mitochondrion functioning, and inter- and intracellular signaling, thus inducing apoptosis (Glabe, 2006).

The protein alteration begins with changes in their secondary structure (Dobson, 2003). This results from destabilization of the macromolecule conformation caused by local changes in the intra- and intercellular media, which may be induced by various external factors (Shemetov et al., 2012). The structural stability is regained through the formation of amyloid-like fibrils consisting of organized associations of modified protein molecules (Go, 1984). This is a two-step process: at the first step, small amyloid oligomers are formed, and at the second step, they assemble into fibrils (Chiti and Dobson, 2006; Glabe, 2006). Protein molecule domains abnormally enriched with β-sheets serve as the primary foci of aggregation. Several hypotheses have been suggested about the origin of these foci. First, the domains that are normally folded, mostly α-helix structures, may be unfolded due to the influence of external factors, such as the extremely low pH or high temperature, to fold again into β-sheets (Nguyen et al., 1995). Second, the protein domains that are normally unfolded may form β-folds (Pawar et al., 2005; Abelein et al., 2012). Third, the stage of a completely unfolded molecule may be skipped, β-sheets arising directly from another folded conformation. In this case, only small regions of the macromolecule are unfolded because of local destabilization, which initially leads to aggregation of the locally altered protein monomers. This, in turn, results in further conformational changes and, finally, formation of amyloid-like protofibrils (Chiti and Dobson, 2009).

It should be noted that precisely the water-soluble oligomeric pre-fibrillar structures are most prone to trigger the mechanisms of cell damage and apoptosis (Glabe, 2006), whereas the protofibrillar and fibrillar structures, being insoluble, exhibit much less cell and tissue toxicity (DeMarco and Daggett, 2004).

Neurodegenerative disorders, including Parkinson's and Alzheimer's diseases (PD and AD, respectively), have been found to be proteinopathies. Proteinopathic alterations have been found in various human and animal tissues, as well as in fungi and prokaryotes, e.g., *Escherichia coli* (de Groot et al., 2009). They may occur in peptides and proteins with different structures and functions. No amino acid sequence has been identified as mandatory for a proteinopathy to develop (Pastor et al., 2007), although larger protein macromolecules have been found to provide better conditions for the formation of abnormal β-folded domains and the resultant proteinopathy (Zhang et al., 2000). Proteinopathies may affect both extracellular and intracellular proteins. For example, heavy-chain amyloidosis is accounted for by accumulation of immunoglobulin aggregations, and PD is related to intracellular aggregation of α-synuclein (Giasson et al., 2003).

Here, we analyzed human insulin aggregation as a model of nanoparticle-induced proteinopathy. Insulin is a hormone secreted by pancreatic β-cells and controlling the blood content of glucose. It is a relatively low-molecular-weight water-soluble protein dimer, its monomers attached to each other via two disulfide bonds. Insulin dimers have been found to associate with one another under physiologically normal conditions to form α-helical hexamers capable of binding Zn^2+^ cations (two or four per hexamer) (Chang et al., 1997; Xu et al., 2012). Experiments using small-angle X-ray scattering analysis (Vestergaard et al., 2007) have demonstrated that these three-dimer associations may give rise to the formation of long fibrils. The secondary structure of insulin in these fibrils largely varies depending on the medium composition and other factors, as evidenced by TEM and FTIR-spectroscopy data. Importantly, not only β-sheet, but also α-helix may be the predominant conformation of the fibrillated protein (Nielsen et al., 2001a). It has been hypothesized (Nielsen et al., 2001b; Ahmad et al., 2003) that insulin fibrillation starts with dissociation of native insulin hexamers into monomers, after which equilibrium is established between unfolded monomers and a partly folded form, whose subsequent oligomerization results in fibrillation nuclei. This assumption agrees with the hypothesis that insulin fibrillation is mediated by its partly unfolded form (Chiti and Dobson, 2009). Furthermore, recent data indicate that insulin could form fibril superstructures through lateral alignment of individual fibrils (Babenko et al., 2011;Babenko and Dzwolak, 2013).

Amyloid-like fibers of insulin protein have been identified in type II diabetic patients (Pease et al., 2010). Insulin molecules also tend to refold and aggregate on arterial walls and membrane surfaces *in vivo*. The aggregation of insulin followed by formation of amyloid-like structures is one of the main problems in insulin production and storage, as well as delivery (Sluzky et al., 1991; Nielsen et al., 2001b). Agitation may play the crucial role in this process (Malik and Roy, 2011).

Nanoparticles, with their unique properties, including bright and stable fluorescence, small size, and capacity for binding capture agents, are regarded as promising fluorescent or magnetic labels to be used for detection of proteinopathies (Georganopoulou et al., 2005). Nanoparticles have also been assumed to interact with amyloidogenic peptides and proteins, thus affecting their fibrillation. This effect is promoted by the high surface-to-volume ratio of nanoparticles, high free Gibbs energy of the interaction, and tunable electrical charge. In this connection, it was suggested that nanoparticles may be used for preventing the aggregation of amyloid-prone peptides and proteins or even inducing disaggregation of amyloid fibrils. For example, maghemite nanoparticles have been found to inhibit the fibrillation of insulin (Skaat et al., 2009a,b). Nanoparticles of various types have been tested to select the most promising candidates, with special focus on a rapid natural clearance after their interaction with protein aggregates (Xiao et al., 2010). On the other hand, a number of nanoparticles have been shown to exert the opposite effect, i.e., facilitate amyloid nucleation by absorbing peptides and proteins, thereby increasing their local concentration (Linse et al., 2007). In the latter case, the nanoparticles may play the role of the centers of protein pseudo-crystallization provoking their further fibrillation.

Of special interest is to study how semiconductor QDs affect the insulin structure in a solution under the conditions corresponding to those in the internal environment of the human body. Owing to their bright, narrow-band fluorescence (Vokhmintcev et al., 2016), QDs have been paid special attention in terms of using them as tags for diagnostic nanoprobes (Brazhnik et al., 2015) and biosensors (Artemyev et al., 2009) in various fields of medicine and biology and as carriers for *in vivo* targeted delivery in animal experiments (Bilan et al., 2016; Ramos-Gomes et al., 2018). It has been found that QDs coated with organic ligands can inhibit the aggregation of amyloid beta (Aβ) peptides forming amyloid plaques in AD. Specifically, CdTe QDs with a shells of thioglycolic acid (TGA) (Yoo et al., 2011) and N-acetyl-L-cysteine (Skaat et al., 2009b) can suppress the fibrillation of the Aβ(1-40) peptide, and those with a dihydrolipoic acid shell inhibit the fibrillation of Aβ(1-42) (Thakur et al., 2011).

Our study has demonstrated important roles of the QD size and surface charge in the perturbation of the insulin protein secondary structure and its subsequent fibrillation under physiological conditions. The remarkable increase in the rate of amyloid-like fibrillation was observed when the protein was treated with QDs of 12 nm hydrodynamic diameter coated with PEG-OH derivative, providing slightly negative charge (−6 mV) for the QD surface. Strongly negative or slightly positive PEG-modified QDs of the same specific diameter or QDs of bigger or smaller diameters had no effect on insulin fibrillation.

Although it is known that human insulin may form amyloid fibers *in vivo* and *in vitro* under extreme environmental conditions (Bucciantini et al., 2002; Jiménez et al., 2002), the capability of nanoparticles to induce insulin fibrillation under physiological conditions was not known up to now. It is worth mentioning that the mechanism of QD-induced insulin fibrillation under physiological conditions may differ from those described for insulin fibrillation under extreme conditions (such as low pH, high temperatures, or strong agitation) described earlier. In any case, the important role of insulin in various biological processes and the presence of this protein in different biological fluids and tissues emphasize the necessity to identify the mechanism of its oligomerization and fibrillation in the presence of nanoparticles in order to find the way to the control of amyloidosis proteinopathies involving this and similar proteins.

## Materials and Methods

Human recombinant insulin protein, ThT, methanol, chloroform, sodium phosphate dibasic, sodium phosphate monobasic, sodium hydroxide, poly-L-lysine, and DL-cysteine hydrochloride hydrate were purchased from Sigma-Aldrich, US. The PEG derivatives HS-C11-EG6, HS-C11-EG6-NH_2_, and HS-C11-EG6-OCH2-COOH were purchased from ProChimia Surfaces Sp, Poland.

### Preparation of a Human Insulin Protein Solution to Study the Fibrillation Process

Dehydrated human insulin protein was dissolved in 0.01 M HCl to obtain the stock solution with a concentration of 20 mg/ml. Then, aliquots of the stock solution were diluted with different buffers to obtain experimental solutions with a concentration of 2 mg/ml (3.44 μM). These human insulin protein preparations were incubated alone or with CdSe/ZnS QDs at different temperatures varying from 25 to 50°C.

### Synthesis and Solubilization of CdSe/ZnS Quantum Dots

CdSe/ZnS QDs were synthesized according to the procedure adapted from that described earlier (Sukhanova et al., 2004). Briefly, two solutions were prepared, one containing 10 g of trioctylphosphine oxide (TOPO, Aldrich) and 5 g of hexadecylamine (HDA, Fluka), and the second containing 80 mg of elemental Se and 110 μl of dimethyl cadmium (Strem, 97%) in 1 ml of trioctylphosphine (TOP, Fluka). The first solution was dried, degassed under vacuum at 180°C, purged with argon, and heated to 340°C under argon flow. Then, fast (<1 s) injection of the second solution into the first one yielded CdSe cores approximately 2 nm in size. Further growth of CdSe cores to the desired size (and, hence, the desired fluorescence color) was induced by prolonged refluxing of the solution at 280°C. After completion of the process, CdSe cores were precipitated at 60°C with methanol, washed twice with methanol, and dried. In order to grow an epitaxial ZnS shell on the CdSe core, a powder of CdSe cores was dissolved in a mixture of 10 g of TOPO and 5 g of HDA. Once again, the mixture was dried and degassed under vacuum at 180°C and purged with argon. A solution containing 210 μl of hexamethyldisilthiane (Fluka) and 130 μl of diethyl zinc (Strem, 97%) in 2 ml of TOPO was added dropwise to this mixture at 220°C under argon flow and intense stirring. The resultant colloidal solution of CdSe/ZnS NPs was slowly cooled to 60°C, and QDs precipitated with methanol were washed twice with methanol and dried. The synthesized QDs contained a CdSe core 2.3 nm (green fluorescence color), 3.1 nm (orange), or 3.9 nm (red) in diameter and an epitaxial shell of several ZnS monolayers (Table 1).

**Table 1:** Physicochemical properties of CdSe/ZnS QDs emitting fluorescence at 530, 570, or 610 nm, coated with PEG derivatives containing terminal groups with different charges.

**Sample^a^**	**Maximum of emission (nm)**	**Core diameter (nm)^b^**	**Hydrodynamic diameter (nm)^c^**	**ζ-potential (mV)^c^**
QD530-PEG-OH	533	2.3 ± 0.2	9.0 ± 1.0	−4.2 ± 0.2
QD570-PEG-OH	570	3.1 ± 0.3	12.0 ± 1.5	−6.0 ± 0.3
QD570-PEG-COOH	570	3.1 ± 0.3	12.0 ± 1.5	−36.0 ± 2.0
QD570-10%PEG-NH_2_/90%PEG-OH	570	3.1 ± 0.3	12.0 ± 1.5	+6.0 ± 0.3
QD610-PEG-OH	610	3.9 ± 0.3	15.0 ± 2.0	−8.9 ± 0.4

a *For all measurements, QD concentrations of ~0.5–1.5 μM were used. In all cases, the absorbance at the first exciton band was <0.10*.

b *QD core average diameters were calculated using transmission electron microscopy images of the corresponding CdSe cores as an average of diameters of nearly 100 nanoparticles*.

c *QD average hydrodynamic diameters and zeta potentials were measured using a Malvern Nano-ZS device as described in section UV-Vis Absorption and Fluorescence Spectroscopy. Presented results are an average of measurements made in triplicate*.

The QDs were solubilized in water using a procedure similar to that published earlier (Sukhanova et al., 2012). Briefly, QDs were first transferred to water after the attachment of DL-cysteine (Sigma) to their surface. The resultant water-soluble QDs displayed a bright green (533 nm), orange (570 nm), or red (610 nm) photoluminescence (PL) with a quantum yield close to 40% at room temperature. Then, DL-cysteine on the surface of QDs was replaced with thiol-containing PEG derivatives with carboxyl or hydroxyl group at the end of the polymer chain; or with a mixture of 10% of thiol-containing PEG derivatives with amino group and 90% of thiol-containing PEG derivatives with hydroxyl groups. Briefly, 156 μl of a 150 mg/ml hydroxy-PEG solution in water, or 138 μl of a 150 mg/ml carboxy-PEG solution in water, or a mixture of 25 μl of a 100 mg/ml amino-PEG and 140 μl of a 150 mg/ml hydroxy-PEG solutions were added to 1 ml of 10 mg/ml preparations of DL-Cys QDs in pure water. The samples were incubated overnight at +4°C, pre-cleaned by centrifugation with Amicon Ultra-15 filter units with a 10 kDa cut-off (Millipore), and finally purified from excess of ligands by gel exclusion chromatography on home-made Sephadex-25 (Sigma) columns.

The described procedure have allowed us to prepare nanoparticles of different diameters and controlled surface charges that are water-soluble and stable in aqueous buffer solutions (Table 1).

### QD Stability Analysis

QD solutions were prepared in 10 mM sodium phosphate buffers (pH 6.0, 7.0, or 8.0). These QD solutions were incubated at different temperatures (25, 37, or 50°C) and analyzed two times a day during the next 2 weeks using the dynamic light scattering analysis, absorption spectral analysis, and fluorescence spectral analysis techniques.

### Insulin Secondary Structure Analysis

Secondary structure analysis was carried out by measuring CD spectra with a Jasco J815 CD spectrometer at 10°C. All measurements were carried out in solutions using a 1 mm path length cell (Hellma). The samples were diluted ten times in the initial buffer. Each spectrum is an average of 20 measurements.

### UV-Vis Absorption and Fluorescence Spectroscopy

UV-Vis absorption spectra were recorded with a Jasco V630Bio spectrophotometer. Fluorescence spectra were obtained using a Jasco FP6600 spectrofluorimeter. Both UV-Vis absorption and fluorescence measurements were obtained employing quartz cells with a 1 cm optical path length.

### ThT Fluorescence Assay

A 10 μM solution of ThT (Sigma) was prepared in 10 mM sodium phosphate buffer at pH 7.0. Thirty microliter aliquots of human insulin protein were taken after different periods of incubation and mixed with 300 μl of a 10 μM solution of ThT. The ThT fluorescence was measured at 482 nm in the semi-micro quartz cell with a 1 cm optical path length at the excitation wavelength of 440 nm.

### Mean Average Diameter and Zeta Potential Measurements

QD average hydrodynamic diameters were measured at different temperatures (25, 37, or 50°C) using the dynamic light scattering (DLS) technique by means of a Malvern Nano-ZS device (Malvern Instrument Ltd., UK). The colloidal stability of the samples was evaluated as a function of time in a sodium phosphate buffer solution at different pH values (pH 6.0, 7.0, or 8.0) after incubation of the samples at 25, 37, or 50°C.

The zeta potential was determined by means of the same device; it was calculated from the electrophoretic mobility using the Smoluchowsky relationship and approximation. The electrophoretic determination of the zeta potential was made at a moderate electrolyte concentration. Zeta-potential measurements of QD solutions were carried out at 25, 37, or 50°C in a sodium phosphate buffer solution with pH 6.0, 7.0, or 8.0.

### Transmission Electron Microscopy

A 10 μl sample was applied onto a copper grid with carbon mesh 200 and pre-treated for 10 min with 1 mg/ml poly-L-lysine. After that, the grid was placed into a 1.5 ml test tube and centrifuged for 5 min at 750 g to remove residual solution. Then, the sample was contrasted with 2% uranyl acetate for 40–50 s. An image was obtained with a JEOL-2100F transmission electron microscope (Jeol Ltd., Japan) at an accelerating voltage of 200 kV. The data were recorded using the DigitalMicrograph™ imaging software (Gatan Inc., USA).

### High-Speed Atomic Force Microscopy Measurements

AFM topography images were recorded using a self-built high-speed atomic force microscope (HS-AFM) setup based on a RIBM equipment (Research Institute of Biomolecule Metrology Co., Ltd, Ibaraki, Japan) (Ando et al., 2001). Experiments were performed at room temperature in tapping mode using silicon nitride cantilever with a spring constant of ~0.2 N m−1 (BL-AC10DS-A2, Olympus). An amorphous carbon layer was grown on the original tip through electron-beam deposition and then sharpened by plasma etching offering an apex of ~4–5 nm. The cantilever's free oscillation amplitude was set at 1–2 nm and the set-point amplitude was ~85% of the free oscillation amplitude so to avoid strong interactions between tip and samples. All measurements were made in imaging buffer (10 mM sodium phosphate, pH 7.2). For observations, a droplet of 2 μL was deposited on freshly cleaved mica. After 5 min of incubation at room temperature, samples were rinsed with imaging buffer. Images were treated with the RIBM software with a flatten filter. Height profiles were used to accurately determine particles size.

The fibril length after each time of incubation was estimated for nearly 120 objects using segmented line measurement by means of the ImageJ software (https://imagej.nih.gov/ij/).

## Results and Discussion

### Interaction of CdSe/ZnS QDs With Human Insulin *in vitro* Followed by Dynamic Light Scattering Measurements and High-Speed Atomic Force Microscopy

CdSe/ZnS core/shell QDs with the CdSe cores 2.3, 3.1, and 3.9 nm in diameter (fluorescing at 533, 570, and 610 nm, respectively) were coated with three-functional PEG derivatives containing terminal OH- (PEG-OH), COOH- (PEG-COOH), or NH_2_- (PEG-NH_2_) groups (Table 1) as described in section Materials and Methods. These procedures yielded batches of well-characterized water-soluble QDs of the same hydrodynamic diameter (12 nm) with slightly negative (−6 mV, QD570-PEG-OH), slightly positive (+6 mV, QD570-10%PEG-NH_2_/90%PEG-OH), or strongly negative (−36 mV, QD570-PEG-COOH) surface charges, as well as batches of slightly negative QDs with smaller (9 nm, QD530-PEG-OH) and larger (15 nm, QD610-PEG-OH) hydrodynamic diameters (Table 1). These panels of QDs have been used in our research for studying human insulin fibrillation induced under physiological conditions by the nanoparticles with different but precisely controlled sizes and surface charges.

**Figure 1:**
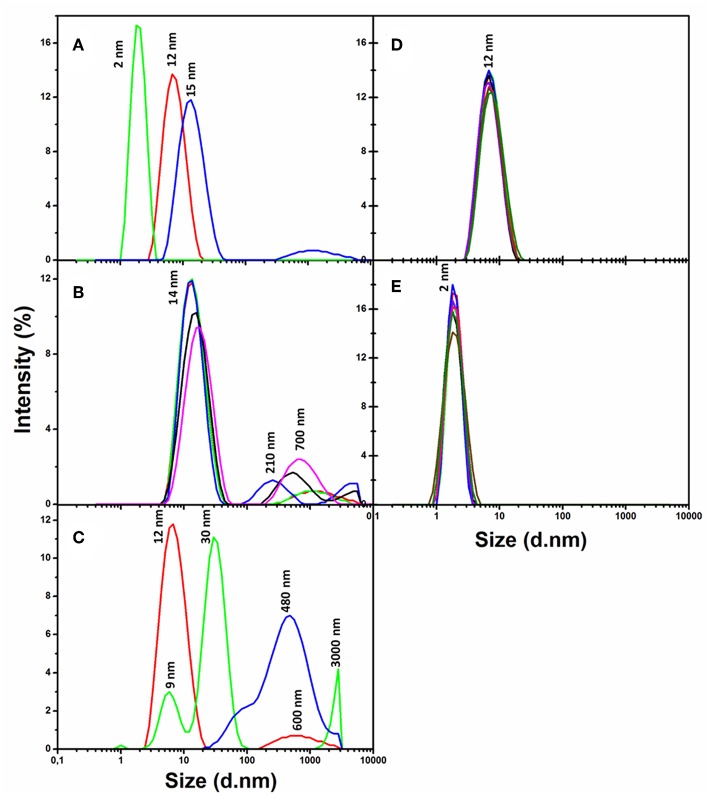
Variation of the size distributions of nanoparticles, human insulin, and nanoparticle–insulin complexes as estimated from the dynamic light scattering (DLS) spectra. **(A)** The DLS spectra of insulin protein alone (green), PEG-OH-modified CdSe/ZnS QDs with a core diameter of 3.1 nm (Table 1) alone (red), and a freshly prepared mixture of insulin with QDs (blue) recoded immediately after mixing of insulin and the QDs. Human insulin (2 mg/ml) was incubated in the presence of QDs (3.44 μM) in a 10 mM sodium phosphate buffer solution (pH 7.0) at 37°C. **(B)** The same QD–insulin mixture as in **(A)** where the DLS spectra were recorded after 0 (red), 5 (green), 10 (blue), 20 (black), and 30 min (magenta) of incubation. **(C)** The same QD–insulin mixture as in **(A)** where the DLS spectra were recorded after 0 (red), 12 (green), and 24 h (blue) of incubation. **(D)** Control experiment: a QD solution (3.44 μM) alone was incubated during 7 days in a 10 mM sodium phosphate buffer solution (pH 7/0) at 37°C. DLS spectra were recorded after 0 (red), 1 (green), 2 (blue), 3 (black), 4 (violet), 5 (rose), 6 (brown), and 7 (dark green) days of incubation. **(E)** Control experiment: a human insulin solution (2 mg/ml) alone was incubated in a 10 mM sodium phosphate buffer solution (pH 7.0) at 37°C, and the measurements were done at the same time points as in **(D)**.

Dynamic light scattering (DLS) analysis (Figure 1) has shown that the slightly negative CdSe/ZnS QDs carrying the PEG-OH derivative on the surface (QD570-PEG-OH, Table 1) remained preserved during more than a week in a phosphate buffer solution (pH 7/0) at 37°C (Figure 1D). Nor did a human insulin solution in the same buffer exhibit any sign of aggregation during 1 week at the same temperature and pH (Figure 1E). DLS analysis also demonstrated that the QDs 570 nm coated with the PEG-COOH derivative (QD-PEG-COOH) or with a mixture of the PEG-OH and PEG-NH_2_ derivatives (QD-PEG-OH/PEG-NH_2_) were also sufficiently stable (data not shown). The stabilities of QD and QD-insulin solutions were constantly controlled by recording their DLS and UV-Vis absorption and fluorescence spectra.

Co-incubation of QD-PEG-OH and recombinant human insulin was found to induce association between insulin and QDs: as seen from the DLS spectra of a freshly made QD-insulin mixture, it contained particles larger than the particles detected in both original solutions of insulin and QD-PEG-OH (Figure 1A). The absorption and fluorescence spectra in the UV-Vis region showed that the QDs in the QD-insulin mixture also remained stable under physiologically normal conditions for more than a week. Thus, the QDs with a PEG-OH shell facilitate the aggregation of human insulin at pH 7.0 and a temperature of 37°C, with the aggregate size rapidly increasing, so that aggregates several micrometers in size appeared within 24 h of co-incubation (Figure 1C). In contrast, co-incubation of QD-PEG-COOH or QD-PEG-OH/PEG-NH_2_ (Table 1) with insulin under the same conditions does not cause noticeable insulin aggregation (data not shown).

Estimation of the changes in the sizes of aggregates in the QD-insulin mixture showed their rapid growth followed by the decrease of originally formed smaller complexes. The QD-induced aggregation of insulin occurred in several stages. At the early kinetics stage (Figure 1B), the quantity of partly folded intermediates of the pre-aggregated protein was decreased and accompanied by formation of some amount of larger aggregates (200–700 nm). The stage of late kinetics, which lasted for 24 h, ended in the strong decrease of the quantity of initial, small intermediates resulted in appearance of significant quantity of 30-nm, 500–1,000 nm, and even 3-μm and larger aggregates (Figure 1C). We assume that the 30-nm pre-aggregates were fibrillation nuclei and 500-nm and bigger particles were amyloid-like fibrils. This assumption agrees with the pathway of the fibril formation out of globular proteins described earlier (Chiti and Dobson, 2009), where partly unfolded forms of proteins aggregated into oligomers, which underwent structural rearrangements resulting in fibrils. Human insulin were earlier reported to form amyloid-like fibrils (Bouchard et al., 2000; Nielsen et al., 2001b), but those studies were performed under extreme acidity (pH 2.0) and temperature (60–70°C) conditions. We have found no available published data on insulin fibrillation under physiologically normal conditions. However, our experiments (Figure 1) have shown that QDs induce the formation of insulin aggregates under the conditions corresponding to the internal environment of the human body.

**Figure 2:**
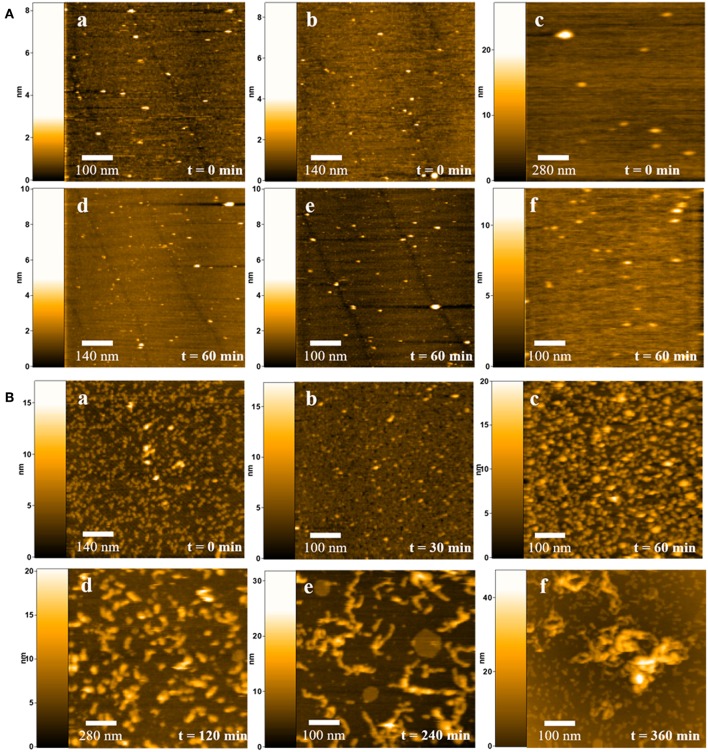
Dynamic AFM imaging of insulin fibrillation in the presence or absence of QDs at physiological conditions. **(A)** Insulin molecules at the zero point of measurement (a) and after 6 h of incubation (d); (b, e) insulin molecules in the presence of PEG-OH at the zero point of measurement (b) and after 6 h of incubation (e); CdSe/ZnS-S-PEG-OH QDs at the zero point of measurement (c) and after 6 h of incubation (f). **(B)** Dynamic AFM imaging of insulin fibrillation in the presence of CdSe/ZnS-S-PEG-OH QDs under physiological conditions for 0.5, 1, 2, 4, and 6 h incubations.

We have also analyzed the time course of QD-induced aggregation of human insulin using high-speed atomic force microscopy (AFM) and compared the results with the DLS data described above. Figure 2 shows AFM images illustrating the formation of insulin fibrils during 6 h of incubation. The formation of the fibrils was analyzed and estimated after 0.5, 1, 2, 4, and 6 h of incubation. Control samples included insulin solution in a 10 mM sodium phosphate buffer (pH 7.2) not containing nanoparticles (Figures 2A, a and d), insulin in a 10 mM sodium phosphate buffer solution (pH 7.2) containing SH-PEG-OH polymer, the free ligand that is on the QD surface (Figures 2A, b and e), and CdSe/ZnS-S-PEG-OH QDs in a 10 mM sodium phosphate buffer solution (pH 7.2) without insulin (Figures 2A, c and f).

We initially estimated the stabilities of CdSe/ZnS-S-PEG-OH QDs alone and insulin alone in a 10 mM sodium phosphate buffer solution (pH 7.2) at 37°C for 6 h. Within this period, no aggregation of CdSe/ZnS-S-PEG-OH QDs or insulin was observed. At the initial moment of time (0 h of incubation), there was no aggregation in the experimental sample, and insulin remained in the form of monomers 18.4 ± 4.56 nm in size. After 30 min of incubation, no noticeable changes occurred in the experimental samples containing CdSe/ZnS-S-PEG-OH QDs; aggregation was not observed (Figure 2B, 30 min). However, after 1 h of incubation of insulin in the presence of CdSe/ZnS-S-PEG-OH QDs, insulin oligomers 22.4 ± 4.46 nm in length were formed (Figure 2B, 1 h). Later, after 2 h of incubation, the insulin oligomers were substantially increased in size, reaching 51.74 ± 8.96 nm (Figure 2B, 2 h). During the next 2 h (after a total of 4 h of incubation), distinct fibrillation was observed, with the length of the fibrils increased to 133.6 ± 17.57 nm (Figure 2B, 4 h), which indicated a high rate of fibril formation. During the last 2 h of incubation, large threadlike fibrils with a length of 175–200 nm were formed (Figure 2B, 6 h).

**Figure 3:**
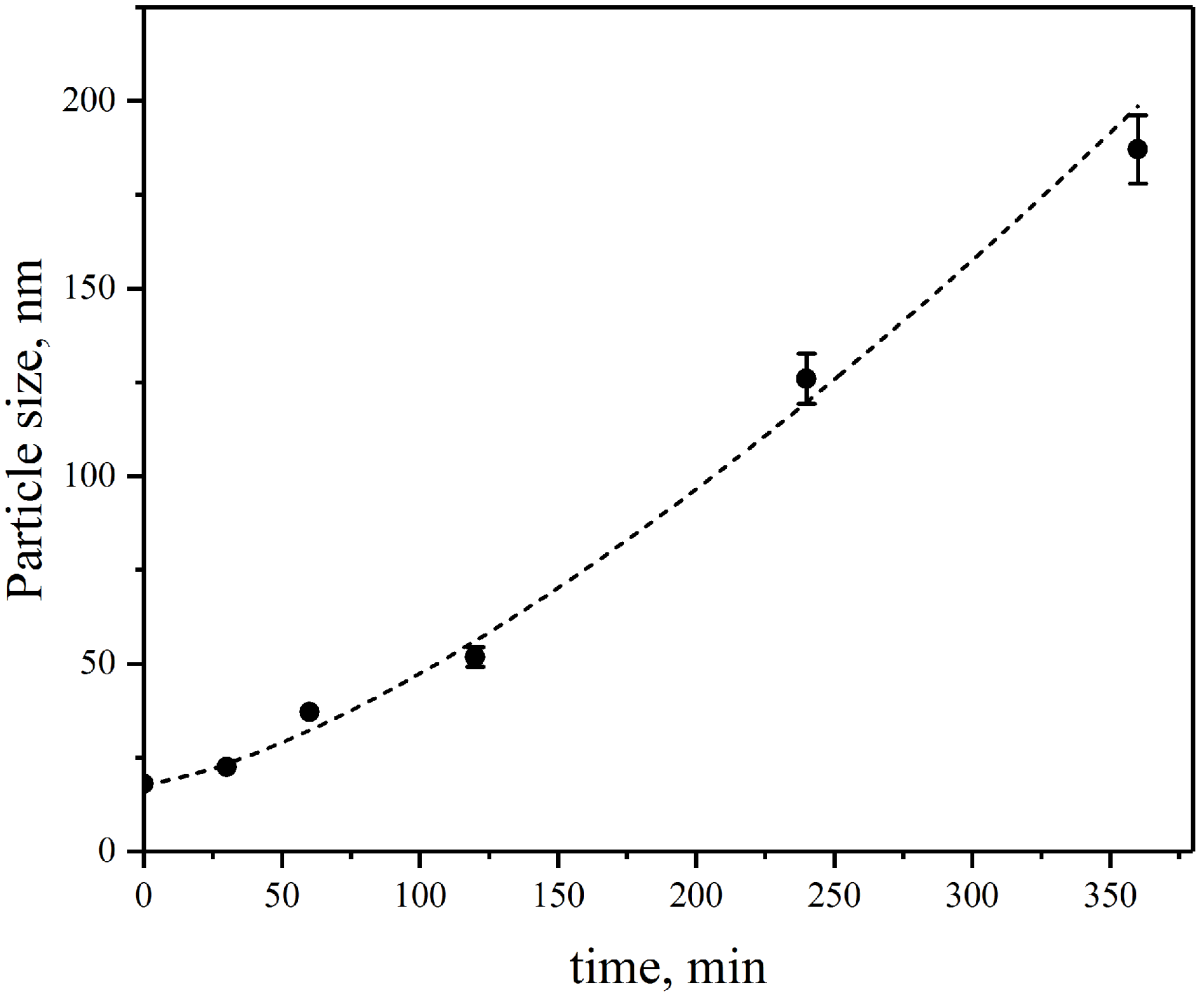
Changes in the sizes of aggregates of insulin during its incubation in the presence of CdSe/ZnS-S-PEG-OH QDs during 0.5, 1, 2, 4, and 6 h of incubation.

Figure 3 shows the summary of the time course of the growth of insulin fibrils in the presence of CdSe/ZnS-S-PEG-OH QDs under the same conditions as that shown in Figure 2 but followed by dynamic light scattering measurements. It is worth noting that no fibrillation was observed in the control insulin solutions not containing nanoparticles or control QD solutions not containing insulin, which suggested that the insulin fibril formation was specifically induced by CdSe/ZnS-S-PEG-OH QDs. The data on the time course of insulin fibrillation in the presence of CdSe/ZnS-S-PEG-OH QDs followed by dynamic light scattering (Figure 3) were closely correlated with the dynamic AFM data (Figure 2): the hydrodynamic size of insulin aggregates was changed from 14 to 140 nm during 4 h of incubation and further to the sizes of nearly 200 nm during the last 2 h of incubation (Figure 3).

We further analyzed the specific molecular structural characteristics of the aggregates formed by the human insulin in the presence of QD-PEG-OH nanoparticles under physiological conditions.

### QD-Induced Insulin Aggregation Is Structurally Similar to Amyloid Fibrillation Phenomena

**Figure 4:**
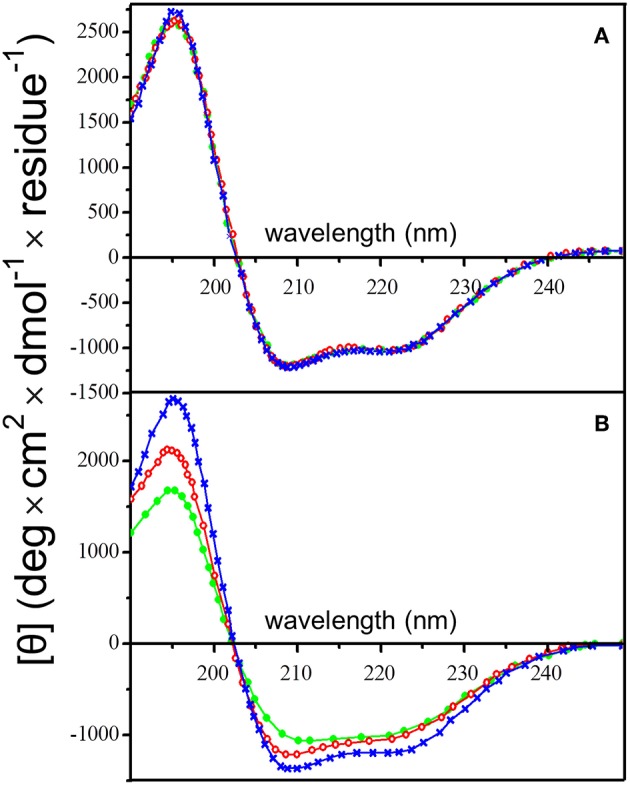
Changes in the insulin secondary structure in the presence of PEG-OH-modified CdSe/ZnS QDs. Circular dichroism spectra of human insulin (2 mg/ml) were recorded **(A)** in the absence or **(B)** in the presence of QDs (3.44 μM). The QDs were also incubated alone (a control sample), when they did not display a noticeable CD signal (baselines in **B**). All solutions were incubated at 37°C for 24 h in a sodium phosphate buffer solution (pH 7.0) at 37°C; the CD spectra were recorded after 0 (blue), 12 (red), and 24 h (green).

Analysis of the circular dichroism (CD) spectra of insulin aggregates resulting from QD-PEG-OH nanoparticle interaction with insulin under the aforementioned conditions indicated structural alterations of insulin corresponding to amyloid-like fibrillation. Signs of this process were observed in the far-UV region of the CD spectrum of insulin (Figure 4). A solution of pure QD-PEG-OH nanoparticles had no CD activity in the UV spectral region (data not shown), whereas a pure insulin solution had a characteristic CD spectrum reflecting the native α-helix-rich secondary structure of this protein, which remained unchanged for 24 h of incubation (Figure 4A). The decrease in the CD intensity at a wavelength of 222 nm upon co-incubation of insulin with the QDs (Figure 4B) indicates a decrease in its α-helix-rich secondary structure. We have calculated the content of insulin secondary structures by deconvolution of its CD spectra using three different approaches: CONTIN-CD, SELCON3, and the recently published BeStSell method (Micsonai et al., 2018). The results obtained by any of these methods did not show significant alpha-to-beta transition upon QD-induced insulin fibrillation. Instead, we observed transformation of nearly 10% of insulin α-helix into unordered or undefined structures. It is worth mentioning that insulin is a low-molecular-weight water-soluble protein dimer. Its monomers are attached to each other via two disulfide bonds and have been found to associate with one another under physiologically normal conditions to form α-helical hexamers capable of binding Zn^2+^ cations (two or four per hexamer) (Chang et al., 1997; Xu et al., 2012). These three-dimer associations may give rise to the formation of long fibrils (Vestergaard et al., 2007) where the secondary structure of insulin largely varies depending on the medium composition and other factors. That is why not only β-sheet, but also α-helix or unordered structures may be the predominant conformation of the fibrillated protein (Nielsen et al., 2001a). Furthermore, some data indicate that insulin could form fibril superstructures through lateral alignment of individual fibrils (Babenko et al., 2011; Babenko and Dzwolak, 2013). Moreover, the mechanism of QD-induced insulin fibrillation under physiological conditions may differ from those described for insulin fibrillation under extreme conditions (such as low pH, high temperatures, or strong agitations) described earlier. In our case, the insulin CD spectra do not indicate strong alpha-to-beta transition upon QD-induced insulin fibrillation under physiological conditions, but show protein unfolding, which may be an indirect indicator of protein secondary structure modifications induced by this process.

In order to investigate the effect of QDs on the fibrillation of human insulin in more specific detail, we monitored the time course of QD-induced insulin fibrillation using the amyloid-specific dye ThT. The fluorophore ThT, as well as some ThT derivatives, has been found to specifically bind with amyloid fibrils and is used for *in vitro* amyloid detection. The quantum yield of the ThT in an aqueous solution is as low as 0.01% (Sulatskaya et al., 2010). Although the fluorescence intensities of ThT bound to amyloid fibrils formed by different amyloidogenic proteins differ significantly, the conclusion that the ThT fluorescence quantum yield increases several orders of magnitude upon dye incorporation into the amyloid fibril is always reasonable (Sulatskaya et al., 2017). It was shown that, in the case of insulin fibrils, the binding of ThT is characterized by the highest binding constants, and the insulin fibril-bound ThT possesses the highest fluorescence quantum yield, reaching 83% (Kuznetsova et al., 2012).

**Figure 5:**
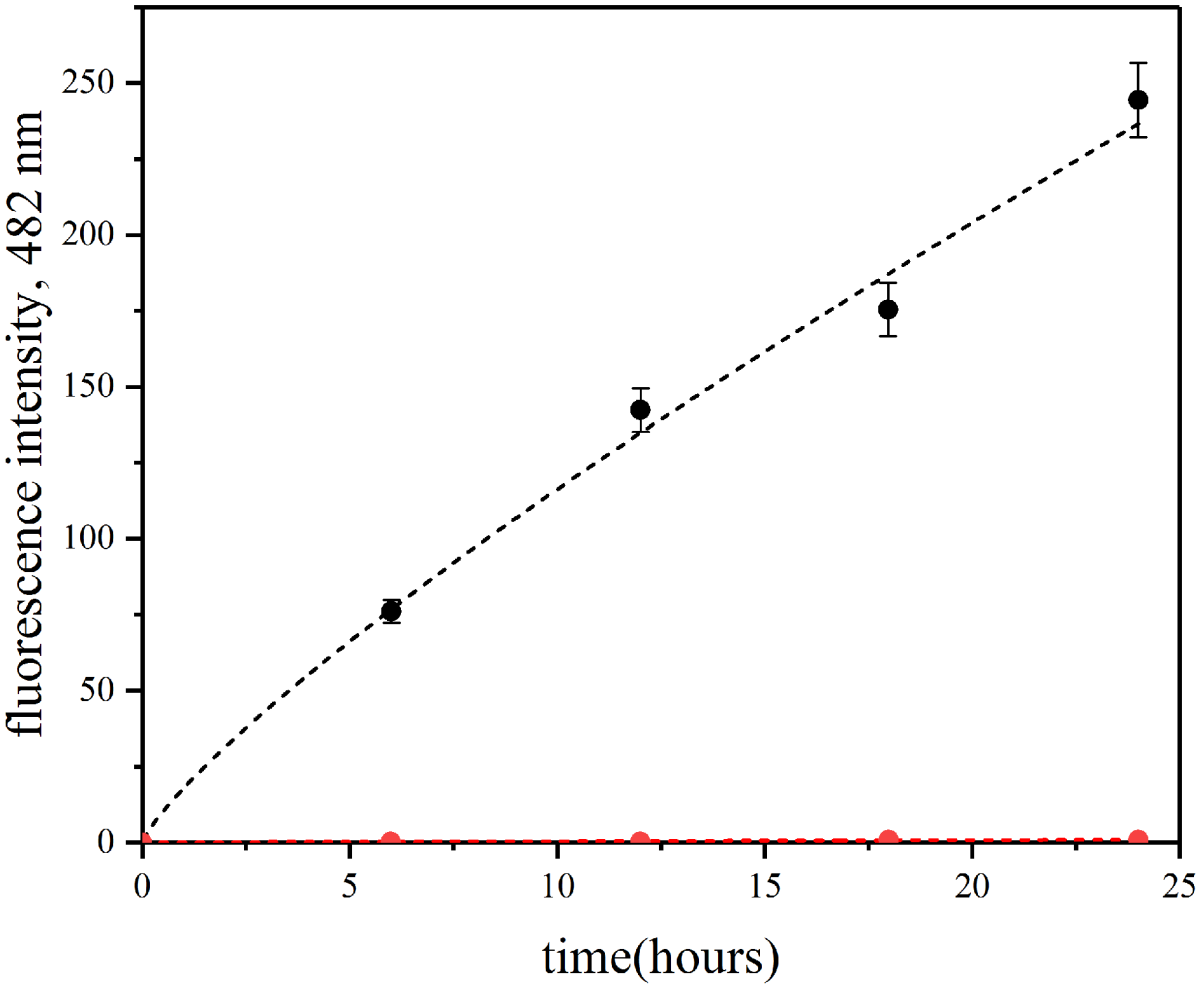
Fibrillation kinetics of insulin at 37°C monitored using the ThT-based fluorescence assay. Human recombinant insulin (2 mg/ml) was incubated in the absence (red) or in the presence (blue) of PEG-OH-modified CdSe/ZnS QDs (3.44 μM) in a 10 mM sodium phosphate buffer solution (pH 7.0) at 37°C for 30 h.

Here, we have used the ThT assay to confirm QD-induced insulin fibrillation in the presence of QD-PEG-OH nanoparticles with a hydrodynamic diameter of 12 nm (Table 1) under physiological conditions. Within 25 h of incubation of insulin (2 mg/ml) in the presence of ThT and without QDs, no fluorescence was detected at a wavelength of 482 nm under the standard conditions (pH 7.0 and a temperature of 37°C) upon 440-nm excitation (Figure 5). Therefore, no amyloid-like fibrils were formed. If the mixture contained QD-PEG-OH nanoparticles, the intensity of ThT fluorescence at 482 nm started to increase and exhibited an almost linear increase during 24 h of incubation, which proved the formation of amyloid-like fibrils (Figure 5).

### Effects of Temperature and pH on QD-Induced Insulin Protein Fibrillation

It has been shown earlier that the native insulin protein may form amyloid fibrils under extreme *in vitro* conditions, namely at a temperature of >60°C and pH <2.37. It is also known that an increase in the incubation temperature or a decrease in pH accelerates insulin fibrillation (Bouchard et al., 2000). Therefore, we analyzed the effects of temperature and pH on the kinetics of insulin fibrillation in the presence of QD-PEG-OH nanoparticles with a hydrodynamic diameter of 12 nm (Table 1) under physiological conditions.

**Figure 6:**
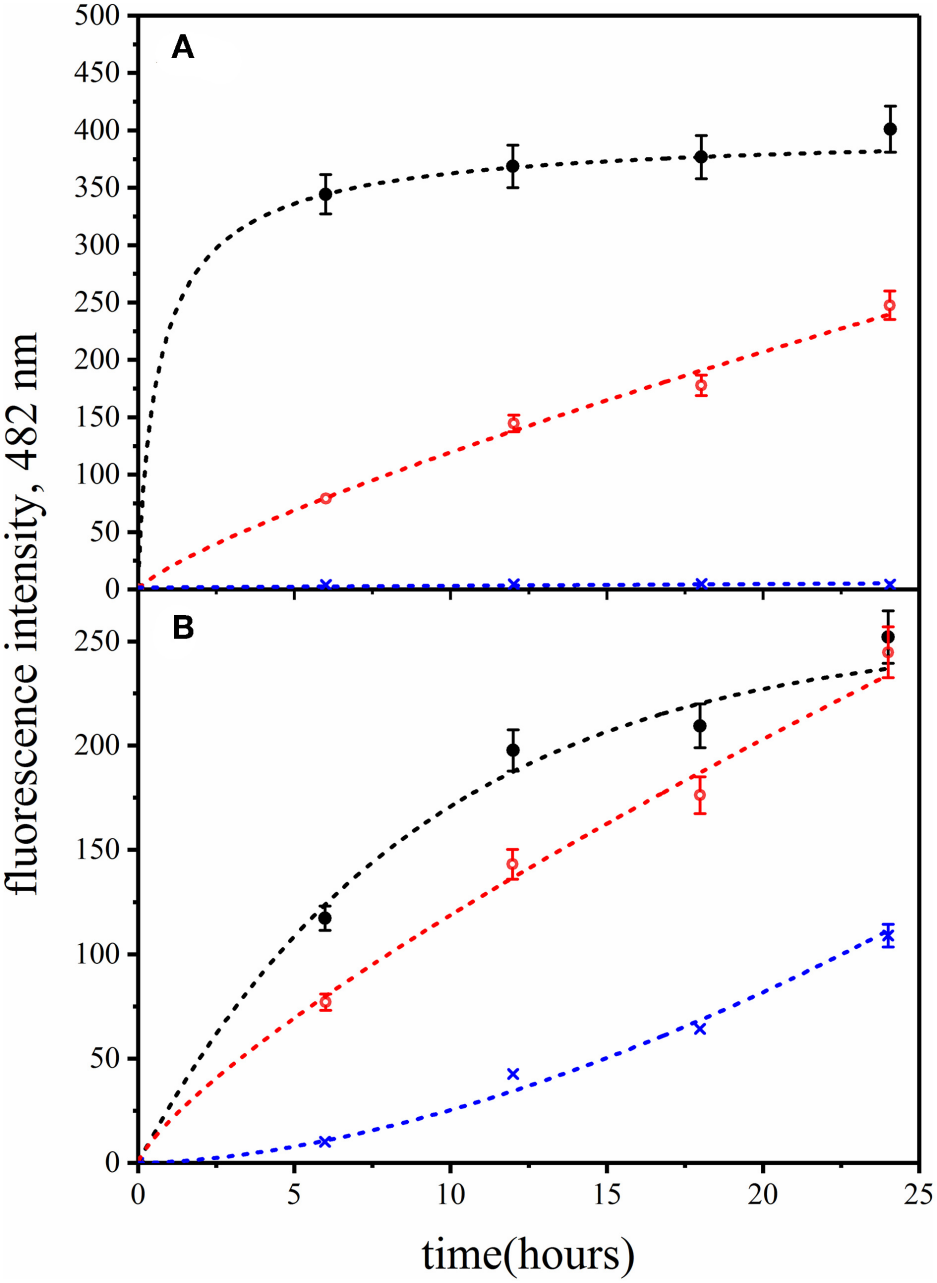
Fibrillation kinetics of insulin at different **(A)** temperatures and **(B)** pH values monitored in the presence of PEG-OH-modified CdSe/ZnS QDs by means of the ThT-based fluorescence assay. Human recombinant insulin (2 mg/ml) was incubated in the presence of PEG-OH-modified CdSe/ZnS QDs (3.44 μM) in a 10 mM sodium phosphate buffer solution (pH 7.0). **(A)** Reaction mixtures were incubated for 24 h at temperatures of 50°C (black), 37°C (red), or 25°C (blue). **(B)** Reaction mixtures were incubated for 12 h at pH 8.0 (black), pH 7.0 (red), or pH 6.0 (blue).

Identical samples of human insulin mixed with QD-PEG-OH nanoparticles were incubated at pH 7.0 and temperatures of 25, 37, and 50°C. The ThT fluorescence assay was used to estimate the influence of temperature variation on the insulin fibrillation rate. Figure 6A shows that there was no noticeable increase in the ThT-specific fluorescence signals after the incubation of the sample at 25°C for 6 h. A rise of the temperature of incubation for the QD-PEG-OH/insulin reaction mixture to 37°C and then to 50°C considerably accelerated insulin fibrillation (Figure 6A). These data show that the QD-induced insulin fibrillation is temperature-dependent, as was previously reported for amyloid-like insulin fibrillation in the absence of QDs (Bouchard et al., 2000; Nielsen et al., 2001b).

It is known that an extremely low pH destabilizes the human insulin protein structure, provoking the alpha-to-beta transition of its secondary structure and fibrillation of the protein (Bouchard et al., 2000). Therefore, we investigated how variation of pH around its physiological values may influence QD-induced insulin fibrillation. Reaction mixtures of human insulin and QD-PEG-OH nanoparticles were prepared in sodium phosphate buffer solutions with pH 6.0, 7.0, or 8.0 and incubated at a temperature of 37°C. QD-induced insulin fibrillation was analyzed using the ThT fluorescence assay.

Figure 6B shows that the rate of QD-induced insulin protein fibrillation was pH-dependent: the rate of the increase in the ThT-specific fluorescence signal was positively correlated with the increase in pH of the incubation buffer solution. In contrast to the literature data (Nielsen et al., 2001b), the rate of insulin fibrillation in the presence of QDs was found to be decreased at lower pH values, whereas an increase in pH caused an increase in the insulin fibrillation rate. This may be due to the influence of pH of the medium on the local ionization of QD surface groups.

This finding supports the hypothesis that the role of QD-PEG-OH nanoparticles in the induction of insulin fibrillation may be related to their action as a pH-specific destabilization agent in a complex manner. The observed specificity of the effect of pH on the insulin protein and its involvement in QD-induced fibrillation led to the assumption that this effect is related to the QD surface charge. Indeed, variation of the local pH affected the charge at the surface of QDs and the QD-insulin interface. In order to test this hypothesis, we studied the relationships between the QD surface charge, QD diameter, and QD-induced amyloid-like fibrillation of insulin.

### Effects of the QD Surface Charge and Diameter on QD-Induced Insulin Fibrillation

In order to analyze the role of the QD surface charge in insulin fibrillation, we compared the characteristics of insulin fibrillation induced by QDs of the same hydrodynamic diameter (12 nm) but coated with different ligands, namely, PEG-COOH or PEG-OH polymer, or a mixture of PEG-NH_2_ and PEG-OH polymers (Table 1), using the amyloid-specific ThT assay. Insulin samples were incubated in the presence of QDs with different coatings at pH 7.0 and 37°C for 24 h. It was shown that only QD-PEG-OH nanoparticles provoked QD-induced fibrillation, providing highly reproducible results, such as those presented in Figure 5, whereas the ThT fluorescence was non-detectable when QD-PEG-NH_2_ or QD-PEG-OH nanoparticles at the same concentrations were used for incubation with the same quantities of insulin. Therefore, only the slightly negatively charged QD-PEG-OH nanoparticles 12 nm in diameter (Table 1) induced insulin fibrillation.

As can be seen in Supplementary Figure 1, neither QD-PEG-COOH nor QD-PEG-OH/PEG-NH_2_ nanoparticles affected the insulin secondary structure, whereas QD-PEG-OH, as it was noted above, altered it by disordering the insulin α-helix structure. The zeta potential values measured at pH 7.0 and 37°C were −6.0 mV for QD-PEG-OH, −36.0 mV for QD-PEG-COOH, and +6.0 mV for QD-PEG-OH/PEG-NH_2_ nanoparticles (Table 1). Thus, the nanoparticles with a slightly negative surface charge most strongly induce human insulin fibrillation. This finding correlates with the earlier data (Wagner et al., 2010) on insulin fibrillation induced by gold nanoparticles, which also had a low negative surface charge.

We have further used QDs of different diameters with the same coating/charge (PEG-OH) in order to vary the curvature of the charged surface interacting with human insulin in a solution. For this purpose, the CdSe/ZnS QDs with hydrodynamic diameters of 9, 12, and 15 nm were used (Table 1). All these QDs were solubilized in water and coated with the same PEG-OH derivative. The zeta potentials of these QDs were measured by the DLS technique in order to estimate the charge on the surface of each particular type of QDs, which yielded the values of −4.2, −6.0, and −8.9 mV, respectively (Table 1). Comparative analysis of the capacity of these QDs for inducing insulin fibrillation during co-incubation in a buffer solution at pH 7.0 and 37°C were analyzed using the ThT amyloid-specific assay. The data showed that only QD-PEG-OH nanoparticles with a hydrodynamic diameter of 12 nm induced fibrillation, yielding highly reproducible results, such as those presented in Figure 5, whereas the ThT fluorescence was non-detectable when QD-PEG-OH nanoparticles 9 or 15 nm in hydrodynamic diameter were incubated with the same concentrations of insulin.

As shown in Supplementary Figure 2, only QD570-PEG-OH nanoparticles with a hydrodynamic diameter of 12 nm induced detectable changes in the insulin secondary structure upon their co-incubation. QD530-PEG-OH (9 nm in diameter) and QD610-PEG-OH (15 nm in diameter) nanoparticles did not induce any observable changes in the insulin CD spectra under the same conditions of incubation; hence, they did not affect the secondary structure of the insulin protein. The differences between the spectra shown in Supplementary Figure 2C can be entirely explained by the noise resulting from the high optical density explained by the larger size of the QD610-PEG-OH nanoparticles.

Supplementary Figure 3 confirms the above conclusion and shows that the kinetics of modification of the insulin secondary structure in the presence of QD-PEG-OH nanoparticles 12 nm in hydrodynamic diameter strongly depends on the QD to insulin molar ratio. Indeed, the data of Supplementary Figure 3 show that progressive increase in the QD concentration in the reaction mixtures where insulin was kept at a constant concentration provokes acceleration of QD-induced modification of the α-helix-rich insulin secondary structure. It is noteworthy that analysis of insulin solution incubated under the same conditions without QD-PEG-OH nanoparticles did not show any signs of modification of the insulin secondary structure during 24 h of incubation at a temperature of 37°C (Figure 4A). Recent data on the influence of CdTe QDs coated with TGA indicate that these nanoparticles inhibit the modification of the secondary structure of amyloidogenic peptide even in the case of a 100-fold excess of Aβ(1-40) over QDs (Yoo et al., 2011). In contrast, in our study, QD-PEG-OH nanoparticles with a hydrodynamic diameter of 12 nm promoted modifications of insulin secondary structure in a concentration-dependent manner. This difference can be explained by the different origin of the functional groups exposed on the QD surface. It is worth mentioning that QDs bearing terminal carboxylic groups had no effect on the insulin secondary structure (Supplementary Figure 2) or protein fibrillation under physiological conditions.

**Figure 7:**
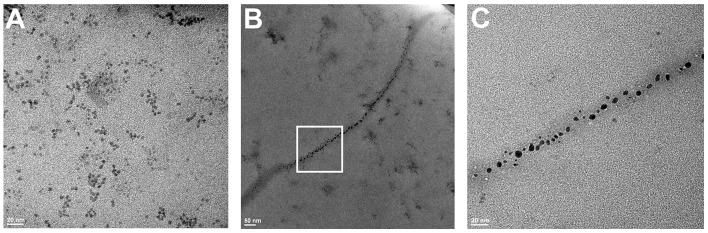
TEM images of QD–insulin mixtures incubated at 37°C. Human recombinant insulin (2 mg/ml) was incubated in presence of PEG-OH-modified CdSe/ZnS QDs (3.44 μM) in a 10 mM sodium phosphate buffer solution (pH 7) at 37°C. The images were obtained after **(A)** 0 and **(B)** 24 h of incubation. **(C)** shows a magnified area framed in a white rectangle in **(B)**.

Finally, in order to directly confirm the formation of fibrillary structures and their association with QDs, we obtained electron micrographs of the samples before and after 24 h of co-incubation of insulin and QD-PEG-OH nanoparticles with a hydrodynamic diameter of 12 nm (Figure 7). Immediately after QD–insulin mixing, there were no ordered structures in the QD-insulin solutions (Figure 7A). After 24 h, fibrils with lengths ranging from 500 to 1,000 nm and an average width of 20–25 nm were formed in the samples (Figures 7B,C). The formation of these fibrils is a hallmark of insulin aggregation and was earlier observed in many studies (Ortiz et al., 2007; Ivanova et al., 2009). It is important that the presence of nanoparticles in the fibrils (Figure 7B) was previously observed only in the case of artificial insulin polymerization in a thin layer containing magnetic nanoparticles (Andersson et al., 2012). Our experiments showed association of QDs and nucleation units of insulin under physiological conditions. These data indicate direct copolymerization of QDs and insulin, with the particles evenly distributed along the fibrils. This confirms that QDs provoke the aggregation of insulin and then remain involved in the process, forming complexes with the intermediates of aggregation.

## Conclusion

Our study has demonstrated that QDs with a specific hydrodynamic diameter (about 12 nm) coated with slightly negative (zeta potential, −6 mV) PEG-OH derivative (Table 1) promote the fibrillation of human insulin under physiological conditions. We have found an important role of the QD size and surface charge in the destabilization of the insulin protein structure and the subsequent insulin fibrillation. Our results confirm that QDs may influence the secondary structure of human insulin and its behavior in a solution. Strongly negative (−36 mV) or slightly positive (+6 mV) PEG-modified QDs (Table 1) of the same hydrodynamic diameter (12 nm), as well as larger (15 nm) or smaller (9 nm) QDs with the same strong negative or small positive charges or a small negative charge (−6 mV) do not promote the fibrillation of insulin under physiological conditions. In contrast, slightly negative (−6 mV) QDs with a hydrodynamic diameter of 12 nm bearing hydroxyl groups on their surface (Table 1) have been shown to strongly accelerate insulin fibrillation.

The finding that insulin fibrillation depends not only on the QD charge, but also on the diameter of QDs and, hence, the size of the charged surface that can interact with a protein molecule and distribution of the charge over this surface due to its different curvature may explain some contradictions between results of similar experiments performed with other proteins and on different classes of nanoparticles (Jiménez et al., 2002; Wu et al., 2008). Apparently, the main, if not the only, reason for these contradictions is that the charge distribution was not taken into account in those studies.

Although it is known that human insulin may form amyloid fibers under extreme environmental conditions (Bucciantini et al., 2002; Jiménez et al., 2002), the capability of nanoparticles to induce insulin fibrillation under physiological conditions has not been known until now. It should be noted that the mechanism of QD-induced insulin fibrillation under physiological conditions may differ from those described for insulin fibrillation under extreme conditions (such as low pH, high temperatures, or strong agitation) described earlier. To determine the protein-specificity of the effect of the surface charge pattern on QD–protein interaction, additional studies are required on the interaction between nanoparticles with different physicochemical properties and amyloid-prone and non-amyloid-prone proteins. If the results of these studies confirm our hypothesis that the surface charge pattern affects the amyloidogenicity of native proteins, then it may be possible to develop “anti-amyloid” nanoparticles exposing a specific pattern of electrical charges on their surface that would modify the *in vivo* conditions in such a way that previously unfolded amyloid-prone proteins refold into their native form. This would not only offer the possibility to decrease the risk entailed in the use of nanoparticles *in vivo* but also pave the way to develop a potent nanoparticle-based tools for the treatment of amyloid-related diseases.

## Author Contributions

AS and IN proposed the concept and designed this study. AS prepared the water-solubilized and stabilized nanoparticles of controlled charge. SP and SB prepared the samples of the insulin-nanoparticle complexes and performed DLS, fluorometric, and CD measurements. ÉL, ME, and MM characterized the complexes and performed high-speed AFM measurements. AS, IN, and AK performed comparative analysis of the results. IN and AS co-wrote the manuscript. All authors commented on the manuscript.

### Conflict of Interest Statement

The authors declare that the research was conducted in the absence of any commercial or financial relationships that could be construed as a potential conflict of interest.
