# Supplementary material for: Nanoparticles With a Specific Size and Surface Charge Promote Disruption of the Secondary Structure and Amyloid-Like Fibrillation of Human Insulin Under Physiological Conditions
Source: Front Chem. 2019 Jul 30;7:480. doi: 10.3389/fchem.2019.00480 (PMC6683663; doi:10.3389/fchem.2019.00480)
Supplement: Supplementary file 1 [file Data_Sheet_1.pdf]

SUPPLEMENTARY MATERIALS

Nanoparticles with a specific size and surface charge promote disruption of the secondary structure and amyloid-like fibrillation of human insulin under physiological conditions

Alyona Sukhanova[1,2,*], Simon Poly[3], Svetlana Bozrova[2], Éléonore Lambert[1], Maxime Ewald[1], Alexander Karaulov[4], Michael Molinari[1], Igor Nabiev[1,2,*]

[1] Laboratoire de Recherche en Nanosciences, LRN-EA4682, UFR de Pharmacie, Université de Reims Champagne-Ardenne, 51 rue Cognacq Jay, 51100 Reims, France

[2] Laboratory of Nano-Bioengineering, National Research Nuclear University MEPhI (Moscow Engineering Physics Institute), 31 Kashirskoe shosse, 115409 Moscow, Russian Federation

[3] Department of Membrane Biophysics, University of Tübingen Interfaculty Institute of Biochemistry, Hoppe-Seyler-Straße 4, 72076 Tübingen Bad-Württemberg, Germany

[4] Department of Clinical Immunology and Allergology, Sechenov First Moscow State Medical University, 119992 Moscow, Russian Federation

*** Correspondence:**

Igor Nabiev or Alyona Sukhanova
igor.nabiev@univ-reims.fr; alyona.sukhanova@univ-reims.fr

Keywords: nanomaterials, protein adsorption, quantum dots, proteinopathies, insulin, fibrillation, amyloidosis, Alzheimer's disease, Parkinson's disease.

Supplementary Figures:

Supp-Fig. 1: Changes in the human insulin secondary structure in the presence of CdSe/ZnS QDs with different surface modification and the same hydrodynamic diameters (12 nm, Table 1).

Supp-Fig. 2: Changes in the human insulin secondary structure in the presence of PEG-OH-modified CdSe/ZnS QDs of different diameters (Table 1).

Supp-Fig. 3: Changes in the human insulin secondary structure in the presence of different concentrations of PEG-OH-modified CdSe/ZnS QDs of 12-nm hydrodynamic diameter (Table 1).

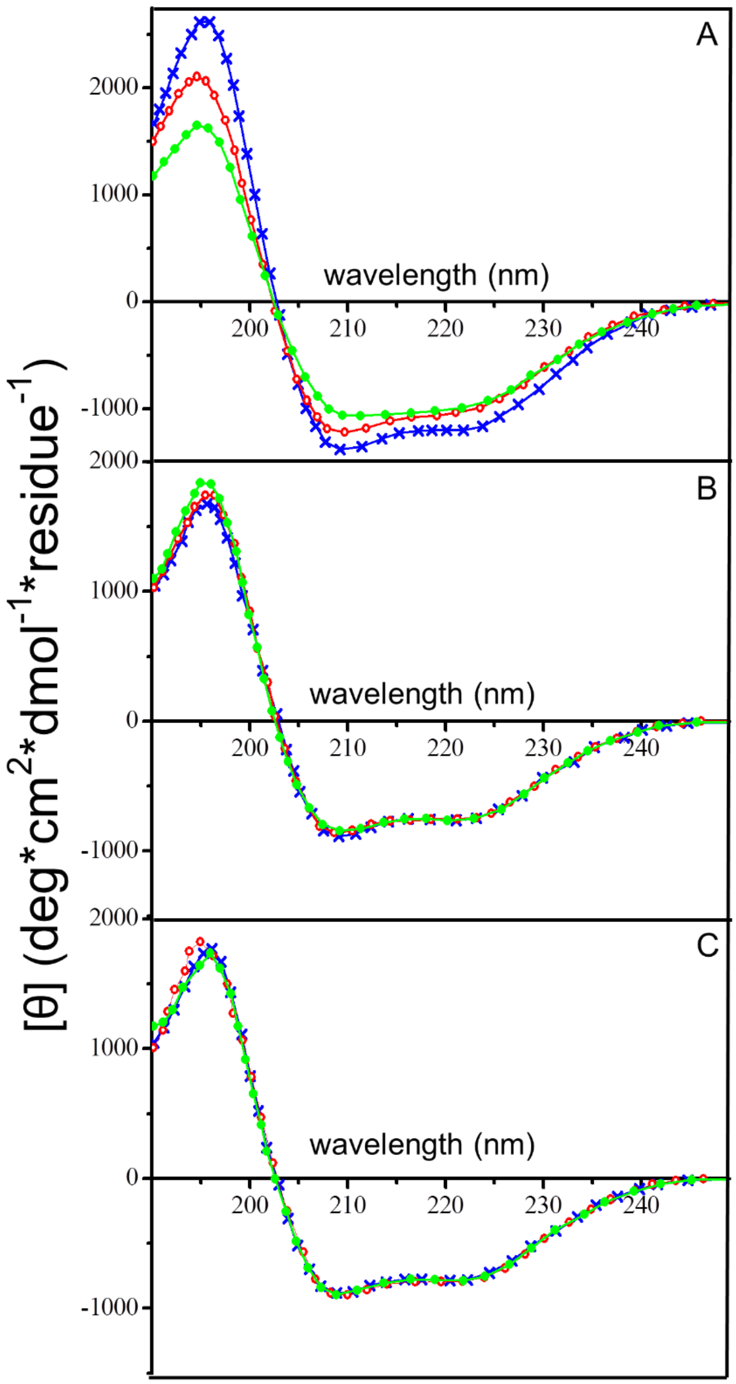

Supp-Fig. 1. Changes in the human insulin secondary structure in the presence of CdSe/ZnS QDs with different surface modification and the same hydrodynamic diameters (12 nm, Table 1).

The circular dichroism spectra of human insulin (2 mg/ml) were recorded in the presence of 3.44 µM of CdSe/ZnS QDs modified with PEG-OH (**A**), PEG-COOH (**B**), and PEG-OH/PEG-NH_2 (**C**). The reaction mixtures were incubated for 24 h in a 10 mM sodium phosphate buffer solution (pH 7) at 37°C, and CD spectra were recorded after 0 (blue), 12 (red), and 24 h (green) of incubation.

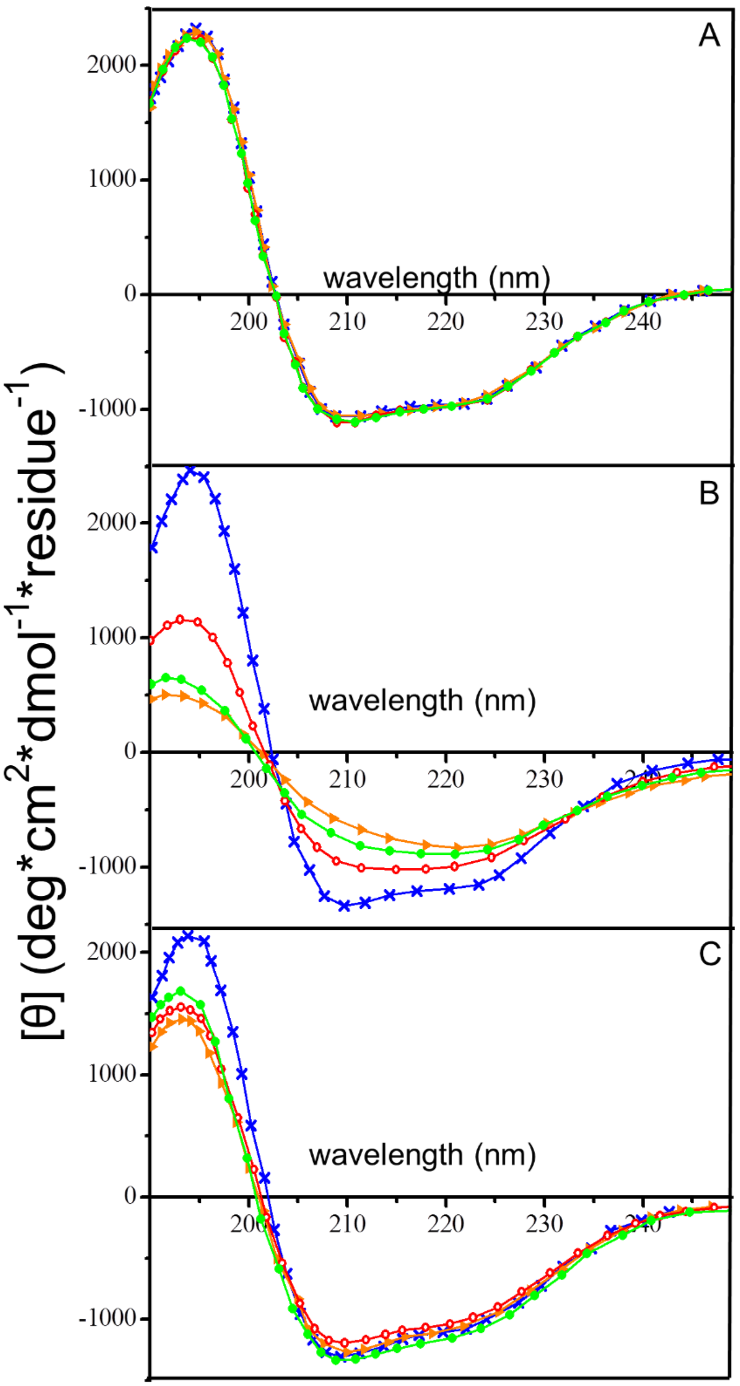

Supp-Fig. 2. Changes in the human insulin secondary structure in the presence of PEG-OH-modified CdSe/ZnS QDs of different diameters (Table 1).

The circular dichroism spectra of human insulin (2 mg/ml) were recorded in the presence of 3.44 µM of QDs with hydrodynamic diameters of 9 nm (**A**), 12 nm (B), or 15 nm (**C**) nm (Table 1).

All solutions were incubated for 3 days in a 10 mM sodium phosphate buffer solution (pH 7) at 37°C, and CD spectra were recorded after 0 (blue), 1 (red), 2 (green), and 3 (orange) days of incubation.

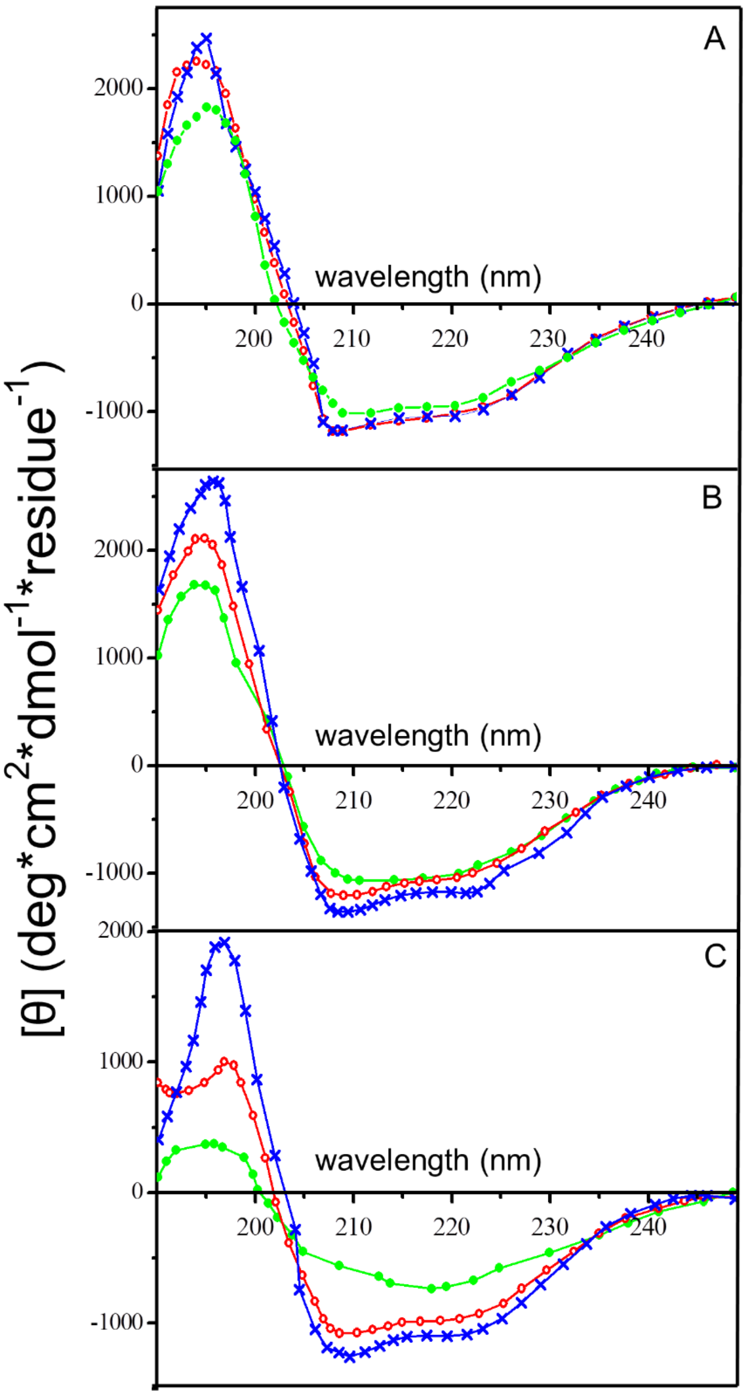

Supp-Fig. 3. Changes in the human insulin secondary structure in the presence of different concentrations of PEG-OH-modified CdSe/ZnS QDs of 12-nm hydrodynamic diameter (Table 1).

The circular dichroism spectra of human insulin (2 mg/ml) were recorded in the presence of different concentrations of QDs: 0.86 μM (**A**), 3.44 μM (**B**), and 13.76 μM (**C**). All solutions were incubated in a 10 mM sodium phosphate buffer (pH 7) at 37°C for 24 h, and CD spectra were recorded after 0 (blue), 12 (red), and 24 h (green) of incubation.
